# Provider perspectives on self-management of hypertension: a survey of perceptions and clinical pharmacist utilization

**DOI:** 10.1186/s12882-025-04731-x

**Published:** 2026-01-24

**Authors:** Cole Howie, Ahmad Al-Masry, Mary K. Good, Patrick Van Eyck, Linder Wendt, Katharine Geasland, Korey Kennelty, Masaaki Yamada, Diana Jalal

**Affiliations:** 1 College of Medicine, The University of Iowa Roy J. and Lucille a Carver, 601 US-6, Bldg 42, Iowa City, IA 52246 USA; 2 Center for Access & Delivery Research and Evaluation, Iowa City VAMC, Iowa City, IA 52246 USA; 3 Institute for Clinical and Translational Science, University of Iowa, Iowa City, IA 52246 USA; 4 Department of Pharmacy Practice and Science, University of Iowa College of Pharmacy, Iowa City, IA 52246 USA

**Keywords:** Hypertension, Clinical pharmacist, Cardiology, Nephrology, Co-management, Medication self-titration, Self-monitoring, Blood pressure control

## Abstract

**Background:**

Previous studies have shown pharmacist-based interventions improve blood pressure (BP) control in individuals with hypertension. Here, we evaluated provider attitudes towards the utilization of at-home self-monitoring of BP, clinical pharmacist support, and pharmacist-guided medication self-titration in the management of hypertension.

**Methods:**

This was a quality improvement project at the University of Iowa Hospitals and Clinics and the Iowa City VA Health Care System. We conducted an electronic survey to determine the attitudes of providers regarding various strategies in the management of hypertension. We surveyed primary care providers, internal medicine residents, nephrologists, cardiologists, nephrology and cardiology fellows, and nurse practitioners. Continuous data were summarized with medians and interquartile ranges and compared across strata using Wilcoxon rank sum tests. Categorical variables were summarized as counts and percentages and compared using Fisher’s exact tests. Analyses were stratified by training status, specialty, and clinical setting. Open-ended comments were analyzed for overarching themes and positive, negative, or neutral sentiment.

**Results:**

Of the 413 surveyed providers, 153 completed the survey, the majority of whom (78%) identified their role as clinicians. We observed high confidence in the diagnosis and treatment of hypertension among all surveyed groups. 91% of providers (*N* = 132) reported that at least once per week or more frequently, their decision to up-titrate BP medications was influenced by a patient’s home BP readings. Nearly half of those surveyed indicated that they had never referred their patients to a clinical pharmacist for BP medication management and a third reported doing so rarely (once or twice a month or less). Respondents, however, agreed that clinical pharmacists could support clinical decision-making for managing BP medications (68%), reduce the burden on providers (68%), and improve the safety of HTN treatment plans (72%).

**Conclusions:**

Clinical pharmacists are underutilized in clinical practice although providers expressed favorable perception of clinical pharmacists’ roles in the management of hypertension. Considering the evidence that pharmacist-based management of high BP improves BP control, interventions are needed to improve pharmacist utilization in the maagement of hypertension.

## Background

Hypertension (HTN) is a widely prevalent disease, estimated to occur in roughly 46% of American adults [1]. Importantly, HTN accounts for more cardiovascular deaths compared to any other modifiable risk factor [2–4], highlighting the critical need for consistent control. Increased prevalence of HTN can be attributed not only to an aging population over time or changes in lifestyle patterns among adults, but also to changes in disease guidelines. The 2017 American College of Cardiology (ACC) and American Heart Association guidelines for HTN decrease diagnostic cutoffs from systolic blood pressure (BP) > 140 mm Hg to 130 mm Hg and/or a diastolic BP > 90 mm Hg to 80 mm Hg [1]. As a result, several studies have shown an increase in the disease prevalence and the need for intensified pharmacotherapies due to suboptimal control based on the current clinical guidelines [5–8]. Heightened demand for HTN management places a significant strain on healthcare systems, particularly on primary care providers (PCPs) and vascular specialists tasked with managing this chronic disease. With mounting pressure on providers to meet such clinical demands, healthcare systems increasingly need innovative strategies to ensure optimized treatment for all patients facing HTN.

A range of available interventions have been shown to improve management of HTN, both individually and when used simultaneously, although challenges to implementation and adherence exist. For example, remote physiologic monitoring (RPM) or remote blood pressure monitoring (RBPM) are billable services that allow for direct communication of remote blood pressure readings to providers. [8] Another innovative strategy is patient self-monitoring of at-home BP, where the patient is provided with a calibrated BP monitor to measure and record daily BP readings. This strategy has been shown to improve BP medication adherence [9] and in-office BP readings [10] and can empower patients to take an active role in managing their own BP. Importantly, more recent findings show that self-monitoring combined with more intensive patient engagement (e.g. pharmacist support, structured education) is most effective in lowering BP in patients with hypertension and hypertension-related comorbidities [11, 12]. Other studies have shown that self-titration, consisting of patients working under the guidance of their provider to make minor adjustments to BP medications, can be safe, feasible, and effective. Taking a self-management approach that combines self-titration with clinical pharmacist supervision and self-monitoring of at-home BP shows promise for increasing patient engagement and improving BP outcomes [13, 14].

Clinical pharmacist integration into physician treatment teams is a solution seen in many clinical practices. Pharmacists bring expertise in medication management, titration, and education that enables them to play a pivotal role in the management of cardiovascular risk factors and diseases [15] including dyslipidemia [16, 17] and heart failure [18–20]. Many studies have shown that pharmacist co-management of hypertension with physicians can lead to reduced blood pressure outcomes [21–24], greater medication adherence [25], higher patient and provider satisfaction [26–28], and greater cost-efficiencies [26, 29, 30]. Prospective studies such as the collaboration among pharmacists and physicians to improve outcome now (CAPTION) trial have demonstrated the effectiveness of physician-pharmacist collaborative models, achieving significant reductions in mean systolic BP (−6.1 mmHg) compared to physician-only care [29–33]. Importantly, patients with additional comorbidities such as diabetes mellitus (DM) or chronic kidney disease (CKD) [34–39] have specifically been shown to benefit significantly from a physician-pharmacist partnership when managing HTN, with expertise in co-managing and optimizing cardio-kidney protective therapies [25]. There is a paucity of data guiding physician-pharmacist collaboration in areas such as at-home BP self-monitoring, the expanded role of clinical pharmacists, and pharmacist-guided self-titration of antihypertensive medications in the United States [40].

We conducted a survey to understand provider perspectives on different approaches to HTN management and their utilization, including self-monitoring of BP, expansion of the clinical pharmacist role, and pharmacist-guided self-titration of BP medication. By identifying gaps in utilization and perceptions of these approaches, this study highlights areas where collaborative care models may be improved with the addition of clinical pharmacist roles in hypertension care.

## Methods

This study was conducted at three clinical settings within a single academic, tertiary-care institution. Clinical settings included clinics within a university hospital (UH), ambulatory clinic (AC), and Veteran Affairs hospital (VA). All three clinics have access to clinical pharmacists in the clinical setting. Clinical pharmacists are physically available at the VA in all clinics. They are available in select clinics at the UH and AC clinic sites. This quality improvement project was determined to be exempt from review, and the need for informed consent was waived, by the University of Iowa Institutional Review Board under the Code of Federal Regulations Title 45 Part 46.104, Office for Human Research Protections. Participation in the survey was voluntary, and survey results were completely anonymous.

Providers at all three settings were surveyed on their attitudes towards management of HTN including self-monitoring, clinical pharmacist involvement, and self-titration. We utilized a 22-item survey originally developed by members of the project team for a separate, previously published study [14]. Survey questions are presented in Table 1. The survey was distributed to our sample using the REDCap online survey data collection platform available at no cost at our institution. Survey participants included learners, comprised of internal medicine residents, cardiology fellows, and nephrology fellows; and non-learner providers, which included physicians within internal medicine serving as PCPs, in addition to specialists, namely cardiology and nephrology. Advanced practice providers within these three fields were included as well. These three fields (internal medicine, cardiology, and nephrology) specifically were chosen for their frequency of HTN diagnosis and management and their expertise in such.

**Table 1:** Survey questions

Provider Characteristics						
1.How many years have you been practicing since receiving your terminal degree or training?	0*	< 5 years		5–9 years	10–14 years	15+ years
2. What is your primary professional role?	Clinician	Clinician educator		Clinician researcher	Clinician administrator	
3 On an average week, how many half day clinic sessions do you spend in clinic?	0	1–2	3–4	5–6	7–8	9+
*Providers that are still completing their terminal training (residency or fellowship).						
**Provider Confidence**						
4. Please rate your confidence in your ability to diagnose hypertension on a scale from 0 to 10, where 0 is not confident at all and 10 is very confident.						
5. Please rate your confidence in your ability to treat hypertension on a scale from 0 - 10, where 0 is not confident at all and 10 is very confident.						
**Practice Frequencies**						
*Please indicate how often you do the following, using a scale of Never, Rarely (once or twice a month or less), Occasionally (Once or twice a week or less), or Frequently (Twice or more per clinic)*						
6. Please indicate how often you initiate antihypertensive treatment for your patients						
7. How often do you up-titrate anti-hypertensives for your patients?						
8. How often do you instruct your patients to monitor home blood pressure and report readings to you or clinic staff?						
9. How often do your patients follow your instructions to monitor home blood pressure and report readings to you or clinic staff?						
10. How often is your decision to up-titrate BP medications influenced by home BP readings?						
11. How often do you refer your patients to the clinical pharmacist for management of BP medications?						
12. How often do you counsel your patients on lifestyle modifications as part of BP management?						
**Statement Opinions**						
*Please indicate the extent to which you agree with the following statements, using a scale of Strongly Disagree, Disagree, Neutral, Agree, Strongly Agree*						
13. Lifestyle and dietary modifications are an integral part of BP management and should be applied routinely in clinical practice						
14. Patient-engagement is important in the management of hypertension						
**Pharmacist-Specific Statement Opinions**						
*Please indicate the extent to which you agree with the following statements, using a scale of Strongly Disagree, Disagree, Neutral, Agree, Strongly Agree*						
15. The pharmacy service supports clinical decision-making for management of BP medications						
16. The pharmacy service improves the safety of treatment plans for patients taking BP medications						
17. The pharmacy service reduces the burden on providers						
18. Pharmacist-guided patient-driven self-titration of BP medications is safe						
19. Pharmacist-guided patient-driven self-titration of BP medications improves BP control						
20. Pharmacist-guided patient-driven self-titration of BP medications improves patient satisfaction						
21. Pharmacist-guided patient-driven self-titration of BP medications improves physician and provider satisfaction						
**Open-Ended Comments**						
22. Please type any comments you have here.						

The survey was distributed to 413 providers across all three clinical sites within the single institution. Provider information such as clinical training and experience were collected via multiple choice questions. Numerical rating scales (0–10) were used for two questions to assess provider confidence in both diagnosing and managing hypertension. All other questions used Likert scales such as frequency-based response options (never, rarely, occasionally, frequently) or agreement response options (strongly agree, agree, neutral, disagree, strongly disagree). These Likert scale questions further assessed provider practices in diagnosing, treating, and optimizing HTN pharmacotherapy. Questions also assessed provider perceptions of clinical pharmacist-guided care on patient outcomes and provider workload.

Continuous data were summarized with medians and interquartile ranges and compared across strata using Wilcoxon rank sum tests. Categorical variables were summarized as counts and percentages and compared using Fisher’s exact tests. Analyses were stratified by training status (learner versus non-learner), specialty (PCP versus subspecialties of cardiology and nephrology), and clinical setting (UH versus AC versus VA). Statistical significance was set at *p* < 0.05.

## Results

Surveys were emailed to 413 providers and 153 were completed, resulting in a 37% response rate. Provider experience and practice characteristics are shown in Table 2. Of 153 responders, 17% (*N* = 26) had less than 5 years of clinical practice, 15% (*N* = 23) had 5–9 years of experience, and 42% (*N* = 65) had 10 or more years of clinical experience. In terms of clinic schedules, 56% (*N* = 85) of all providers identified that they have at least three times a week or more with a half-day clinic, while 33% (*N* = 50) have a half-day clinic once or twice a week.

**Table 2:** Provider experience and practice

Years Since Terminal Training	OVERALL *N*=153	Training Comparison			Specialty Comparison			Clinical Site Comparison			
		Learner *N*=44 (%)	Non-Learner *N*=109 (%)	*p*	PCP *N*=64 (%)	Specialist *N*=19 (%)	*p*	UH *N*=75 (%)	AC *N*=24 (%)	VA *N*=54 (%)	*p*
0	39 (25%)	37 (84.0%)	2 (1.8%)	<0.001	0 (0%)	0 (0%)	>0.9	21 (28%)	4 (17%)	14 (26%)	0.5
< 5	26 (17%)	2 (4.5%)	24 (22.0%)		5 (26%)	12 (19%)		15 (20%)	4 (17%)	7 (13%)	
5-9	23 (15%)	5 (11.0%)	18 (17.0%)		3 (16%)	12 (19%)		13 (17%)	4 (17%)	6 (11%)	
10-14	16 (10%)	0 (0%)	16 (15.0%)		3 (16%)	10 (16%)		8 (11%)	4 (17%)	4 (7.4%)	
15+	49 (32%)	0 (0%)	49 (45.0%)		8 (42%)	30 (47%)		18 (24%)	8 (33%)	23 (43%)	
Primary Role											
Clinician	119 (78%)	42 (95%)	77 (71%)	0.002	9 (47%)	42 (66%)	0.016	56 (75%)	17 (71%)	46 (85%)	0.13
Clinician educator	19 (12%)	0 (0%)	19 (17%)		3 (16%)	16 (25%)		13 (17%)	3 (12%)	3 (5.6%)	
Clinician researcher	11 (7.2%)	2 (4.5%)	9 (8.3%)		6 (32%)	3 (4.7%)		4 (5.3%)	2 (8.3%)	5 (9.3%)	
Clinician administrator	4 (2.6%)	0 (0%)	4 (3.7%)		1 (5.3%)	3 (4.7%)		2 (2.7%)	2 (8.3%)	0 (0%)	
Average half-day clinic per week											
0	18 (12%)	2 (4.5%)	16 (15%)	<0.001	2 (11%)	6 (9.4%)	<0.001	9 (12%)	0 (0%)	9 (17%)	0.002
1-2	50 (33%)	28 (64%)	22 (20%)		12 (63%)	9 (14%)		27 (36%)	6 (25%)	17 (31%)	
3-4	27 (18%)	10 (23%)	17 (16%)		3 (16%)	12 (19%)		15 (20%)	5 (21%)	7 (13%)	
5-6	24 (16%)	3 (6.8%)	21 (19%)		0 (0%)	19 (30%)		12 (16%)	5 (21%)	7 (13%)	
7-8	19 (12%)	1 (2.3%)	18 (17%)		1 (5.3%)	12 (19%)		10 (13%)	7 (29%)	2 (3.7%)	
9+	15 (9.8%)	0 (0%)	15 (14%)		1 (5.3%)	6 (9.4%)		2 (2.7%)	1 (4.2%)	12 (22%)	
	OVERALL Median (IQR*)	Learner Median (IQR)	Non-Learner Median (IQR)		PCP Median (IQR)	Specialist Median (IQR)		UH Median (IQR)	AC Median (IQR)	VA Median (IQR)	
Confidence in ability to diagnose HTN	9.0 (9.0, 10.0)	8.0 (8.0, 9.0)	10.0 (9.0, 10.0)	<0.001	10.0 (9.0, 10.0)	10.0 (9.0, 10.0)	>0.9	9.0 (9.0, 10.0)	9.0 (9.0, 10.0)	10.0 (9.0, 10.0)	0.08
Confidence in ability to treat HTN	9.0 (8.0, 9.0)	8.0 (7.0, 9.0)	9.0 (8.0, 10.0)	<0.001	9.0 (8.0, 10.0)	9.0 (9.0, 9.25)	0.5	8.0 (8.0, 9.75)	9.0 (8.0, 9.0)	9.0 (8.0, 10.0)	0.4
		Training Comparison			Specialty Comparison			Clinical Site Comparison			
Years Since Terminal Training	OVERALL *N*=153	Learner *N*=44 (%)	Non-Learner *N*=109 (%)	*p*	PCP *N*=64 (%)	Specialist *N*=19 (%)	*p*	UH *N*=75 (%)	AC *N*=24 (%)	VA *N*=54 (%)	*p*
0	39 (25%)	37 (84.0%)	2 (1.8%)	<0.001	0 (0%)	0 (0%)	>0.9	21 (28%)	4 (17%)	14 (26%)	0.5
< 5	26 (17%)	2 (4.5%)	24 (22.0%)		5 (26%)	12 (19%)		15 (20%)	4 (17%)	7 (13%)	
5-9	23 (15%)	5 (11.0%)	18 (17.0%)		3 (16%)	12 (19%)		13 (17%)	4 (17%)	6 (11%)	
10-14	16 (10%)	0 (0%)	16 (15.0%)		3 (16%)	10 (16%)		8 (11%)	4 (17%)	4 (7.4%)	
15+	49 (32%)	0 (0%)	49 (45.0%)		8 (42%)	30 (47%)		18 (24%)	8 (33%)	23 (43%)	
Primary Role											
Clinician	119 (78%)	42 (95%)	77 (71%)	0.002	9 (47%)	42 (66%)	0.016	56 (75%)	17 (71%)	46 (85%)	0.13
Clinician educator	19 (12%)	0 (0%)	19 (17%)		3 (16%)	16 (25%)		13 (17%)	3 (12%)	3 (5.6%)	
Clinician researcher	11 (7.2%)	2 (4.5%)	9 (8.3%)		6 (32%)	3 (4.7%)		4 (5.3%)	2 (8.3%)	5 (9.3%)	
Clinician administrator	4 (2.6%)	0 (0%)	4 (3.7%)		1 (5.3%)	3 (4.7%)		2 (2.7%)	2 (8.3%)	0 (0%)	
Average half-day clinic per week											
0	18 (12%)	2 (4.5%)	16 (15%)	<0.001	2 (11%)	6 (9.4%)	<0.001	9 (12%)	0 (0%)	9 (17%)	0.002
1-2	50 (33%)	28 (64%)	22 (20%)		12 (63%)	9 (14%)		27 (36%)	6 (25%)	17 (31%)	
3-4	27 (18%)	10 (23%)	17 (16%)		3 (16%)	12 (19%)		15 (20%)	5 (21%)	7 (13%)	
5-6	24 (16%)	3 (6.8%)	21 (19%)		0 (0%)	19 (30%)		12 (16%)	5 (21%)	7 (13%)	
7-8	19 (12%)	1 (2.3%)	18 (17%)		1 (5.3%)	12 (19%)		10 (13%)	7 (29%)	2 (3.7%)	
9+	15 (9.8%)	0 (0%)	15 (14%)		1 (5.3%)	6 (9.4%)		2 (2.7%)	1 (4.2%)	12 (22%)	
	OVERALL Median (IQR*)	Learner Median (IQR)	Non-Learner Median (IQR)		PCP Median (IQR)	Specialist Median (IQR)		UH Median (IQR)	AC Median (IQR)	VA Median (IQR)	
Confidence in ability to diagnose HTN	9.0 (9.0, 10.0)	8.0 (8.0, 9.0)	10.0 (9.0, 10.0)	<0.001	10.0 (9.0, 10.0)	10.0 (9.0, 10.0)	>0.9	9.0 (9.0, 10.0)	9.0 (9.0, 10.0)	10.0 (9.0, 10.0)	0.08
Confidence in ability to treat HTN	9.0 (8.0, 9.0)	8.0 (7.0, 9.0)	9.0 (8.0, 10.0)	<0.001	9.0 (8.0, 10.0)	9.0 (9.0, 9.25)	0.5	8.0 (8.0, 9.75)	9.0 (8.0, 9.0)	9.0 (8.0, 10.0)	0.4

Percentages calculated with denominator representing the number of respondents versus total survey participants due to missing data in various survey questions

IQR: Inter-quartile Range

**Fig. 1:**
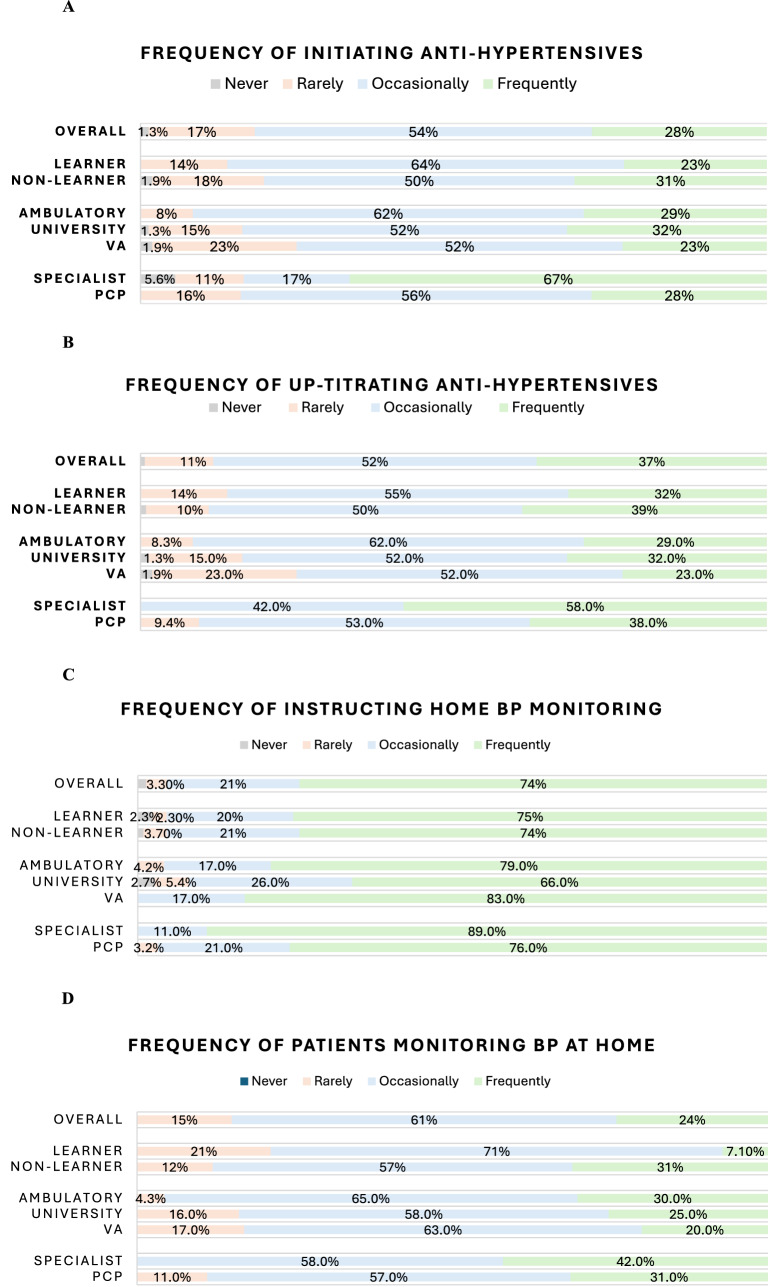

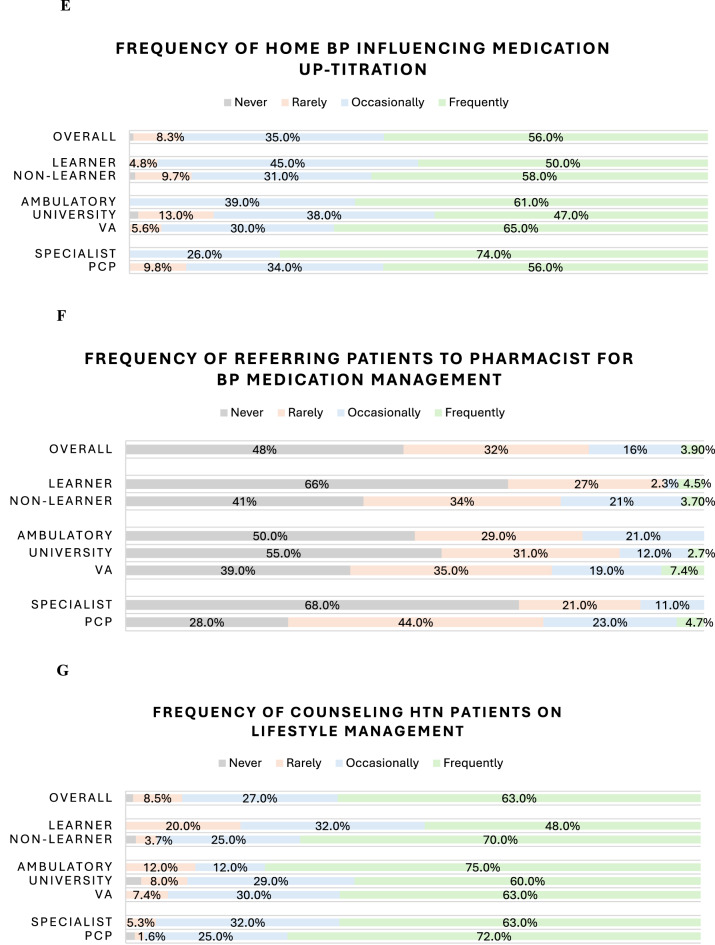
Likert scale data on frequency of practices survey questions for providers

Reported average confidence in both diagnosing and treating hypertension was high at 9 out of 10 (10 being the most confident). The majority of providers reported they initiate BP medications (Fig. 1A) and up-titrate medications (Fig. 1B) at least once a week. 91% of providers (*N* = 132) reported up-titrating BP medications based on the patient’s home BP readings at least once per week or more frequently (Fig. 1E). Provider Practice Survey responses are shown in Table 3.

**Table 3:** Provider practice survey question results

		Training Comparison			Specialty Comparison		Clinical Site Comparison				
	OVERALL N=153	Learner N=44 (%)	Non-Learner N=109 (%)	p*	PCPN=64 (%)	SpecialistN=19 (%)	p*	UHN=75 (%)	ACN=24 (%)	VAN=54 (%)	p*
Please indicate how often you intitiate antihypertensive treatment for your patients:				0.5			0.002				0.6
Never	2 (1.3%)	0 (0%)	2 (1.9%)		0 (0%)	1 (5.6%)		1 (1.3%)	0 (0%)	1 (1.9%)	
Rarely (Once or twice a month or less)	25 (17%)	6 (14%)	19 (18%)		10 (16%)	2 (11%)		11 (15%)	2 (8.3%)	12 (23%)	
Occasionally (Once or twice a week or less)	81 (54%)	28 (64%)	53 (50%)		36 (56%)	3 (17%)		39 (52%)	15 (62%)	27 (52%)	
Frequently (Twice or more per clinic)	43 (28%)	10 (23%)	33 (31%)		18 (28%)	12 (67%)		24 (32%)	7 (29%)	12 (23%)	
How often do you up-titrate anti-hypertensives for your patients?				0.8			0.2				0.13
Never	1 (0.7%)	0 (0%)	1 (0.9%)		0 (0%)	0 (0%)		1 (1.3%)	0 (0%)	0 (0%)	
Rarely (Once or twice a month or less)	17 (11%)	6 (14%)	11 (10%)		6 (9.4%)	0 (0%)		9 (12%)	2 (8.3%)	6 (11%)	
Occasionally (Once or twice a week or less)	79 (52%)	24 (55%)	55 (50%)		34 (53%)	8 (42%)		41 (55%)	7 (29%)	31 (57%)	
Frequently (Twice or more per clinic)	56 (37%)	14 (32%)	42 (39%)		24 (38%)	11 (58%)		24 (32%)	15 (62%)	17 (31%)	
How often do you instruct your patients to monitor home blood pressure and report readings to you or clinic staff?				>0.9			0.7				0.2
Never	2 (1.3%)	1 (2.3%)	1 (0.9%)		0 (0%)	0 (0%)		2 (2.7%)	0 (0%)	0 (0%)	
Rarely (Once or twice a month or less)	5 (3.3%)	1 (2.3%)	4 (3.7%)		2 (3.2%)	0 (0%)		4 (5.4%)	1 (4.2%)	0 (0%)	
Occasionally (Once or twice a week or less)	32 (21%)	9 (20%)	23 (21%)		13 (21%)	2 (11%)		19 (26%)	4 (17%)	9 (17%)	
Frequently (Twice or more per clinic)	113 (74%)	33 (75%)	80 (74%)		48 (76%)	17 (89%)		49 (66%)	19 (79%)	45 (83%)	
		Training Comparison			Specialty Comparison		Clinical Site Comparison				
	OVERALL N=153	Learner N=44 (%)	Non-Learner N=109 (%)	p*	PCPN=64 (%)	SpecialistN=19 (%)	p*	UHN=75 (%)	ACN=24 (%)	VAN=54 (%)	p*
How often do patients follow instructions to monitor home blood pressure and report readings to you/clinic staff?				0.003			0.3				0.6
Never	0 (0%)	0 (0%)	0 (0%)		0 (0%)	0 (0%)		0 (0%)	0 (0%)	0 (0%)	
Rarely (Once or twice a month or less)	21 (15%)	9 (21%)	12 (12%)		7 (11%)	0 (0%)		11 (16%)	1 (4.3%)	9 (17%)	
Occasionally (Once or twice a week or less)	88 (61%)	30 (71%)	58 (57%)		35 (57%)	11 (58%)		39 (58%)	15 (65%)	34 (63%)	
Frequently (Twice or more per clinic)	35 (24%)	3 (7.1%)	32 (31%)		19 (31%)	8 (42%)		17 (25%)	7 (30%)	11 (20%)	
How often is your decision to up-titrate BP medications influenced by home BP readings?				0.4			0.3				0.2
Never	1 (0.7%)	0 (0%)	1 (1.0%)		0 (0%)	0 (0%)		1 (1.5%)	0 (0%)	0 (0%)	
Rarely (Once or twice a month or less)	12 (8.3%)	2 (4.8%)	10 (9.7%)		6 (9.8%)	0 (0%)		9 (13%)	0 (0%)	3 (5.6%)	
Occasionally (Once or twice a week or less)	51 (35%)	19 (45%)	32 (31%)		21 (34%)	5 (26%)		26 (38%)	9 (39%)	16 (30%)	
Frequently (Twice or more per clinic)	81 (56%)	21 (50%)	60 (58%)		34 (56%)	14 (74%)		32 (47%)	14 (61%)	35 (65%)	
How often do you refer your patients to the clinical pharmacist for management of BP medications?				0.004			0.023				0.5
Never	74 (48%)	29 (66%)	45 (41%)		18 (28%)	13 (68%)		41 (55%)	12 (50%)	21 (39%)	
Rarely (Once or twice a month or less)	49 (32%)	12 (27%)	37 (34%)		28 (44%)	4 (21%)		23 (31%)	7 (29%)	19 (35%)	
Occasionally (Once or twice a week or less)	24 (16%)	1 (2.3%)	23 (21%)		15 (23%)	2 (11%)		9 (12%)	5 (21%)	10 (19%)	
Frequently (Twice or more per clinic)	6 (3.9%)	2 (4.5%)	4 (3.7%)		3 (4.7%)	0 (0%)		2 (2.7%)	0 (0%)	4 (7.4%)	
How often do you counsel your patients on lifestyle modifications as part of BP management?				0.003			0.5				0.5
Never	2 (1.3%)	0 (0%)	2 (1.8%)		1 (1.6%)	0 (0%)		2 (2.7%)	0 (0%)	0 (0%)	
Rarely (Once or twice a month or less)	13 (8.5%)	9 (20%)	4 (3.7%)		1 (1.6%)	1 (5.3%)		6 (8.0%)	3 (12%)	4 (7.4%)	
Occasionally (Once or twice a week or less)	41 (27%)	14 (32%)	27 (25%)		16 (25%)	6 (32%)		22 (29%)	3 (12%)	16 (30%)	
Frequently (Twice or more per clinic)	97 (63%)	21 (48%)	76 (70%)		46 (72%)	12 (63%)		45 (60%)	18 (75%)	34 (63%)	

Fisher’s Test - Simulated P-Value; Fisher’s exact test; Wilcoxon rank sum test; Pearson’s Chi-squared test

Nearly half (48%, *N* = 74) of all providers surveyed indicated that they had never referred their patients to a clinical pharmacist for BP medication management (Fig. 1F), and 32% (*N* = 49) reported doing so rarely (once or twice a month or less). Respondents, however, agreed that clinical pharmacists could support clinical decision-making for managing BP medications (68%, *N* = 102), reduce the burden on providers (68%, *N* = 103), and improve the safety of HTN treatment plans (72%, *N* = 110). Additionally, 72% (*N* = 108) of all providers surveyed agreed that pharmacist-guided, patient-driven self-titration of BP medications is safe. Responses to all Pharmacist-Specific survey questions are shown in Table 4.

**Table 4:** Pharmacist-Specific survey question results

	OVERALL N=153	Training Comparison			Specialty Comparison			Clinical Site Comparison			
		Learner N=44 (%)	Non-Learner N=109 (%)	p*	PCPN=64 (%)	SpecialistN=19 (%)	p*	UHN=75 (%)	ACN=24 (%)	VAN=54 (%)	p*
The pharmacy service supports clinical decision-making for management of BP medications:											
Strongly Agree	45 (30%)	5 (11%)	40 (37%)	0.012	28 (44%)	2 (11%)	0.013	18 (24%)	10 (43%)	17 (31%)	0.6
Agree	57 (38%)	19 (43%)	38 (35%)		22 (34%)	7 (39%)		31 (41%)	5 (22%)	21 (39%)	
Neutral	41 (27%)	16 (36%)	25 (23%)		10 (16%)	9 (50%)		22 (29%)	7 (30%)	12 (22%)	
Disagree	5 (3.3%)	2 (4.5%)	3 (2.8%)		2 (3.1%)	0 (0%)		2 (2.7%)	1 (4.3%)	2 (3.7%)	
Strongly Disagree	4 (2.6%)	2 (4.5%)	2 (1.9%)		2 (3.1%)	0 (0%)		2 (2.7%)	0 (0%)	2 (3.7%)	
The pharmacy service improves the safety of treatment plans for patients taking BP medications:											
Strongly Agree	52 (34%)	9 (20%)	43 (40%)	0.12	32 (50%)	2 (11%)	0.002	21 (28%)	10 (43%)	21 (39%)	0.6
Agree	58 (38%)	18 (41%)	40 (37%)		20 (31%)	8 (44%)		35 (47%)	6 (26%)	17 (31%)	
Neutral	32 (21%)	13 (30%)	19 (18%)		7 (11%)	8 (44%)		14 (19%)	6 (26%)	12 (22%)	
Disagree	5 (3.3%)	2 (4.5%)	3 (2.8%)		2 (3.1%)	0 (0%)		2 (2.7%)	1 (4.3%)	2 (3.7%)	
Strongly Disagree	5 (3.3%)	2 (4.5%)	3 (2.8%)		3 (4.7%)	0 (0%)		3 (4.0%)	0 (0%)	2 (3.7%)	
The pharmacy service reduces the burden on providers:											
Strongly Agree	46 (30%)	8 (18%)	38 (35%)	0.085	29 (45%)	1 (5.6%)	0.006	17 (23%)	8 (35%)	21 (39%)	0.7
Agree	57 (38%)	16 (36%)	41 (38%)		19 (30%)	11 (61%)		32 (43%)	8 (35%)	17 (31%)	
Neutral	37 (24%)	14 (32%)	23 (21%)		12 (19%)	6 (33%)		20 (27%)	6 (26%)	11 (20%)	
Disagree	7 (4.6%)	3 (6.8%)	4 (3.7%)		2 (3.1%)	0 (0%)		3 (4.0%)	1 (4.3%)	3 (5.6%)	
Strongly Disagree	5 (3.3%)	3 (6.8%)	2 (1.9%)		2 (3.1%)	0 (0%)		3 (4.0%)	0 (0%)	2 (3.7%)	
		Training Comparison			Specialty Comparison			Clinical Site Comparison			
	OVERALL N=153	Learner N=44 (%)	Non-Learner N=109 (%)	p*	PCPN=64 (%)	SpecialistN=19 (%)	p*	UHN=75 (%)	ACN=24 (%)	VAN=54 (%)	p*
Pharmacist-guided patient-driven self-titration of BP medications is safe:											
Strongly Agree	51 (34%)	8 (19%)	43 (40%)	0.1	33 (52%)	2 (11%)	0.006	25 (33%)	10 (43%)	16 (30%)	0.6
Agree	57 (38%)	21 (49%)	36 (33%)		19 (30%)	8 (44%)		27 (36%)	10 (43%)	20 (38%)	
Neutral	34 (23%)	11 (26%)	23 (21%)		8 (12%)	7 (39%)		20 (27%)	2 (8.7%)	12 (23%)	
Disagree	5 (3.3%)	2 (4.7%)	3 (2.8%)		2 (3.1%)	0 (0%)		2 (2.7%)	1 (4.3%)	2 (3.8%)	
Strongly Disagree	4 (2.6%)	1 (2.3%)	3 (2.8%)		2 (3.1%)	1 (5.6%)		1 (1.3%)	0 (0%)	3 (5.7%)	
Pharmacist-guided patient-driven self-titration of BP medications improves BP control:											
Strongly Agree	46 (30%)	9 (20%)	37 (34%)	0.4	28 (44%)	1 (5.6%)	0.002	23 (31%)	8 (35%)	15 (28%)	0.8
Agree	63 (41%)	21 (48%)	42 (39%)		23 (36%)	8 (44%)		29 (39%)	10 (43%)	24 (44%)	
Neutral	40 (26%)	14 (32%)	26 (24%)		11 (17%)	8 (44%)		22 (29%)	5 (22%)	13 (24%)	
Disagree	1 (0.7%)	0 (0%)	1 (0.9%)		0 (0%)	1 (5.6%)		1 (1.3%)	0 (0%)	0 (0%)	
Strongly Disagree	2 (1.3%)	0 (0%)	2 (1.9%)		2 (3.1%)	0 (0%)		0 (0%)	0 (0%)	2 (3.7%)	
Pharmacist-guided patient-driven self-titration of BP medications improves patient satisfaction:											
Strongly Agree	40 (26%)	8 (18%)	32 (30%)	0.084	21 (33%)	3 (17%)	0.2	19 (25%)	6 (26%)	15 (28%)	0.9
Agree	56 (37%)	22 (50%)	34 (32%)		21 (33%)	5 (28%)		29 (39%)	9 (39%)	18 (34%)	
Neutral	52 (34%)	13 (30%)	39 (36%)		19 (30%)	10 (56%)		26 (35%)	8 (35%)	18 (34%)	
Disagree	1 (0.7%)	1 (2.3%)	0 (0%)		0 (0%)	0 (0%)		1 (1.3%)	0 (0%)	0 (0%)	
Strongly Disagree	2 (1.3%)	0 (0%)	2 (1.9%)		2 (3.2%)	0 (0%)		0 (0%)	0 (0%)	2 (3.8%)	
		Training Comparison			Specialty Comparison			Clinical Site Comparison			
	OVERALL N=153	Learner N=44 (%)	Non-Learner N=109 (%)	p*	PCPN=64 (%)	SpecialistN=19 (%)	p*	UHN=75 (%)	ACN=24 (%)	VAN=54 (%)	p*
Pharmacist-guided patient-driven self-titration of BP medications improves physician and provider satisfaction:											
Strongly Agree	45 (30%)	9 (20%)	36 (34%)	0.074	26 (41%)	2 (11%)	0.004	22 (29%)	7 (30%)	16 (30%)	0.6
Agree	58 (38%)	21 (48%)	37 (35%)		24 (38%)	5 (28%)		30 (40%)	10 (43%)	18 (34%)	
Neutral	44 (29%)	12 (27%)	32 (30%)		12 (19%)	11 (61%)		23 (31%)	6 (26%)	15 (28%)	
Disagree	2 (1.3%)	2 (4.5%)	0 (0%)		0 (0%)	0 (0%)		0 (0%)	0 (0%)	2 (3.8%)	
Strongly Disagree	2 (1.3%)	0 (0%)	2 (1.9%)		2 (3.1%)	0 (0%)		0 (0%)	0 (0%)	2 (3.8%)	

Fisher’s Test - Simulated P-Value; Fisher’s exact test; Wilcoxon rank sum test; Pearson’s Chi-squared test

### Learners vs non-learners

Learners made up 29% (*N* = 44) of surveyed providers, while 71% (*N* = 109) were non-learners, including physicians and advanced practice providers. The majority of learners averaged 1–2 half-days of clinic per week (64%, *N* = 28). A small but significant difference was found in the confidence between learners and non-learners in diagnosing and managing hypertension. The average response among learners was an 8 out of 10 (10 being most confident) in confidence in diagnosing hypertension, compared to an average response of 10 among non-learners (*p* < 0.001). Confidence in treating hypertension showed an average response of 8 in learners compared to 9 in non-learners (*p* < 0.001). No differences were observed between these two groups in frequency of treating hypertension. Learners reported lower utilization of self-monitored home BP in clinical practice and lower rates of counseling patients on lifestyle changes (Fig. 1G).

Notably, non-learners had a higher proportion of providers who agreed or strongly agreed that pharmacists supported clinical decision-making for management of BP medications compared to learners (72% vs 54%, respectively; *p* = 0.012). Learner providers were found to report “never” for how often they consulted pharmacy for BP medication management more often than non-learners (66% vs 41%, respectively; *p* = 0.004). Full comparisons between learner and non-learner providers can be found in Tables 2 and 3.

### Primary care providers compared to specialists

We then compared the perspective of the non-learner PCPs and specialty physicians. In our survey, 77% (*N* = 64) identified as PCPs and 23% (*N* = 19) as specialists within cardiology and nephrology (the remaining 26 non-learners were advanced practice providers). Specialists were found to average fewer half-days in clinics per week compared to PCPs. Specialists had a higher proportion of providers that reported initiating BP medications frequently (twice or more per clinic session) compared to PCPs (67% vs 28%, respectively; *p* = 0.002). When comparing attitudes towards pharmacist-led management of hypertension, fewer specialists reported referring patients to pharmacists for aid in BP medication management compared to PCPs. Most specialists reported never referring their patients to pharmacists (68% vs 28% of PCPs; *p* = 0.023). Additionally, a lower proportion of specialists agreed or strongly agreed that pharmacists supported clinical decision-making for BP management compared to PCPs (50% to 79%, respectively; *p* = 0.013), improved safety of treatment plans (55% vs 81%, respectively; *p* = 0.002), or reduced provider burden (67% vs 75%, respectively; *p* = 0.006). When considering the role clinical pharmacists can play in supporting patient self-management, fewer specialists agreed that pharmacist-guided, patient-driven self-titration of BP medications was both safe (55% vs 82%, respectively; *p* = 0.006) and improved outcomes (50% vs 80%, respectively; *p* = 0.002). Just 6% of specialists strongly agreed with the prompt that pharmacist-guided medication titration improved BP control, compared to 44% of PCPs (*p* = 0.002). Additionally, only 39% of specialists agreed with the statement that pharmacist-guided patient-driven self-titration of BP medications improved both physician and patient satisfaction, compared to 79% of PCPs (*p* = 0.004) (See Table 4).

### The impact of practice location

No differences were found in years of clinical experience for respondents at the UH, AC, and VA locations. There was significant site-level heterogeneity in the amount of time providers spent in clinic, as measured across six ordinal response levels (*p* = 0.002). For example, nearly half of VA providers (35%, *N* = 54) reported only one or two half-days of clinic per week, while responses at UH (49%, *N* = 75) and AC (16%, *N* = 24) sites were more evenly distributed across higher time commitments. No differences were found between locations in provider confidence in diagnosing or treating hypertension. Survey responses on frequency of initiating (Fig. 1A), up-titrating (Fig. 1B), or counseling patients on BP monitoring at home (Figs. 1C, 1D) and continued lifestyle changes at home (Fig. 1G) also showed no difference across locations. Importantly, no differences were identified between locations regarding referral to the clinical pharmacists or attitudes towards the clinical pharmacist role. Full comparisons of providers across clinic locations can be found in Table 2.

### Open-ended responses

Respondents also had the opportunity to enter open-ended text comments at the conclusion of the survey. Of the 153 providers who completed the survey, 24% (*N* = 36) provided additional comments. We conducted sentiment and thematic content analyses of providers’ narrative comments to better understand their attitudes and experiences related to pharmacist utilization. Open-ended comments were coded inductively and separately grouped by positive, negative, or neutral sentiment by the research team. Responses were also grouped by provider type to assess differences in knowledge/awareness, attitudes, and experience with clinical pharmacist programs.

From the 36 narrative comments, 8 were discarded due to lack of relevance (e.g., commenting on personal clinic schedules, structure of the survey itself, etc.). The majority (60%, *N* = 17) of the remaining 28 responses communicated positive sentiment regarding clinical pharmacist utilization, while only 11% (*N* = 3) of responses were negative and 29% (*N* = 8) were neutral in sentiment. Positive-sentiment comments detailed experiences working successfully with clinical pharmacists or commented on their ability to help manage patient loads, while negative comments mainly focused on patient safety concerns. Neutral comments mainly expressed a lack of knowledge or experience in working with clinical pharmacists. In both negative and neutral sentiment categories, comments reflected a possible lack of understanding regarding clinical pharmacists’ roles and abilities.

**Fig. 2:**
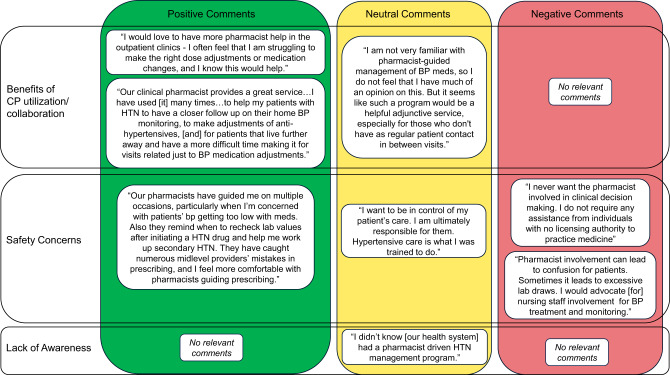
Examples of Open-ended comments a range of representative comments from survey respondents are shown here arranged by the three main thematic categories (top to bottom: benefits of cp utilization/collaboration, safety concerns, and lack of awareness) and showing positive, Neutral, and negative comments from left to right

Three main themes were identified among the responses: Benefits of clinical pharmacist utilization and collaboration; Safety concerns; and Lack of awareness. Respondents emphasized the *benefits of clinical pharmacist utilization and collaboration* in many open-ended comments, reporting very positive experiences working with clinical pharmacists and that they were considered essential to patient care. Additionally, they pointed out that pharmacist involvement could enhance quality of care and frequency of patient contact while decreasing provider burden. Some providers brought up *safety concerns* in relying on clinical pharmacists given patients’ health literacy and capacity for involvement in their own care. Additionally, a small number of providers perceived pharmacist involvement as taking some control of patient care away from the clinicians ultimately responsible for the patient. Providers also acknowledged that they needed more information about working with pharmacists or did not have very much experience with pharmacists in managing hypertension, signaling a *lack of awareness* and a need for more information regarding how clinical pharmacists could support patient care. See Fig. 2 for examples of responses for each theme.

## Discussion

This study aims to evaluate the preferences and practices of providers treating hypertension in settings with clinical pharmacist support. These findings reveal high confidence in diagnosing and treating hypertension by providers across the board, from trainees to advanced practice providers and attending physicians regardless of clinical setting. With the majority of respondents having reported initiating (82%) or up-titrating (89%) BP medications for patients once a week or more, it is clear that a high demand exists for hypertension management support. The reported high frequency of up-titrating BP medications based on at-home BP self-monitoring overall (91% once a week or more frequently) suggests that providers support this strategy for HTN management. Additionally, the majority of surveyed providers of any type agreed that pharmacists involved in co-management help improve treatment safety (72% of providers agreeing or strongly agreeing), supporting clinical decision-making (67%), and reducing provider burden (67%). Open-ended comments from respondents also show that many providers recognize the benefits of pharmacist support for those that had experience with this collaboration. Despite these widely-held beliefs, nearly half of all providers surveyed had never utilized this resource, revealing a gap between perceptions and practice. Underutilization was observed even at the VA site where access to clinical pharmacists is widely available through the Patient Aligned Care Team (PACT) model; no differences were found between sites in usage (see Table 3).

Our findings highlight an underutilized resource in hypertension care despite the growing evidence supporting the positive impact of pharmacist-led interventions [21–24, 29]. Studies such as the CAPTION trial [29] have shown significant reductions in blood pressure and improved outcomes, including among minority patients. Other trials have demonstrated the cost-effectiveness of pharmacist intervention in diverse settings, including urban community environments [30–33]. Patient and provider satisfaction with pharmacists co-managing HTN has also been well documented in previous studies. Multiple studies have shown that patients who have HTN co-managed by both physician and pharmacist have higher satisfaction ratings and preference for this treatment modality compared to physician-only treatment groups [26, 27]. Interviewed veteran patients participating in a physician-pharmacist co-managed medication program highlighted preference for having such a team of experts to rely on for assistance, education, and faster results, particularly when pharmacists are able to help with dose titration of medications outside of the physician clinic [27]. When pharmacists provided comprehensive medication management in partnership with physician-owned clinics, surveyed patients were found to have higher satisfaction and higher levels of comfort with pharmacist co-management [41]. This same study also found high physician satisfaction as well, with PCPs reporting improvements in their patients’ cardiovascular risk factors with helpful pharmacists’ recommendations. Several other studies have also verified PCP satisfaction [28, 32, 42], with one study finding 90% of physicians agreeing that pharmacists in their clinics made medication management more efficient [43].

Beyond traditional co-management models, emerging evidence supports pharmacist-guided self-monitoring and self-titration as a promising strategy to enhance HTN care in a more efficient manner. Randomized trials such as telemonitoring and self-management in the control of hypertension (TASMINH2) and targets and self-management for the control of blood pressure in stroke and other at-risk groups (TASMIN-SR) have demonstrated significant reduction in BP through the use of patient-driven medication titration when paired with structured protocols and clinical oversight [13, 44]. While former trials focus on the general population, we initiated a randomized controlled trial that extends to veterans with CKD, using pharmacist-led education and individualized titration plans to enable patients to safely adjust their antihypertensive therapies as warranted [45]. Our novel approach has the potential to empower patients, reduce clinical inertia, and alleviate provider workload particularly in team-based care settings such as those seen already at Veteran Health Administration health systems.

While the benefits of pharmacist involvement are well-documented, our findings suggest that barriers to implementation remain, particularly among specialists who reported lower utilization of the clinical pharmacists and lower favorability of the pharmacist role in the management of hypertension. Several factors could explain the divergence of opinions and utilization of pharmacists for HTN control between PCPs and specialists. The additional costs of incorporating pharmacists into practice settings as well as the barriers to pharmacists being granted prescribing authority or ability to bill for services could represent significant challenges to widespread utilization of pharmacists in healthcare systems. However, some teams and clinics use Medicare billing codes for coordination of care events like Chronic Care Management (CCM) or Transitional Care Management (TCM) to bill for the services pharmacists provide as part of the care team. [46] Additionally, some teams and clinics justify funding of pharmacists through showing their contributions to value-based payments or cost avoidance.

Another possibility lies in specialists’ inability to integrate pharmacists into their care teams. Previous reports from the ACC in 2009 have highlighted significant underutilization of nonphysician practitioners, including pharmacists, and suggest that cardiologists often lack familiarity with the optimal integration of nonphysician professionals into their care teams [47]. A recent study comparing pharmacist care models found that clinical pharmacists increased from 16% in traditional referral-based models to 81% in co-visit settings [48], where clinical pharmacists and physicians see patients together during the same visit. This suggests that our observed rate of 26% represents a gap between evidence-based best practices and real-world implementation. When viewed through this implementation science lens, clinical pharmacists remain underutilized in hypertension care.

Another possible factor for the lower utilization of clinical pharmacists by physician specialists may lie in the higher level of confidence in HTN management expressed by the specialists. Consistently, specialists at our institution reported initiating new HTN medications significantly more frequently in clinic compared to their PCP colleagues. As such, specialists may feel that additional support from pharmacists is unnecessary for their clinical decision-making and might not have considered the ways pharmacists could assist in adjusting dosages and answering questions after the specialist encounter. Variation in levels of understanding of clinical pharmacists’ expertise and abilities may influence specialists’ perceptions of the clinical pharmacist role. Open-ended responses indicated that some providers were uncertain about allowing pharmacists to make adjustments to patient medications or did not understand what role pharmacists could play. Other responses from providers who had prior assistance from pharmacists with medication management in other conditions, such as encountered in primary care, reported that these experiences led them to believe this would be beneficial in managing hypertension. Taken together, these findings indicate that increasing awareness of the roles that pharmacists could play in HTN management and education on the benefits of pharmacist collaboration could increase trust and utilization of this approach.

While much of the literature emphasizes the benefits of interdisciplinary team-based care, it is important to acknowledge potential concerns raised by both PCPs and specialists. From the PCP and patient perspective, some studies have reported concerns about fragmented care and diffusion of responsibility in shared-care models, particularly in systems lacking robust communication and coordination protocols [49]. Similarly, our paper is one of the first to explicitly highlight the relatively low referral rates to the clinical pharmacist specialists by physician specialists. Despite positive perceptions of pharmacists’ capabilities, barriers such as unfamiliarity with their scope, concerns about added complexity, and possible discomfort with direct pharmacist follow-up contact may deter referrals. Anecdotal accounts also suggest that professional boundary concerns and outdated perceptions of pharmacists as primarily dispensing roles may continue to hinder collaborative practice [50]. Addressing these perceptions through interprofessional education, defined roles, and streamlined communication workflows may help build confidence in pharmacist collaboration.

Regardless of physician preferences, there may be external forces at play that prevent physicians from being able to utilize pharmacists’ pharmacologic expertise in HTN management decision-making. The incorporation of pharmacists into a physician’s clinical practice can represent increased resource utilization and cost. For example, one randomized control trial found a significant increase in clinic visits for HTN patients managed in the physician-pharmacist intervention arm versus the physician-only arm (7.2 vs 4.9 visits per patient, respectively; *p* < 0.0001) [22]. Other studies have found that pharmacist service reimbursement and sustainability can be an issue at some institutions, despite the proven cost-effectiveness of pharmacist involvement in clinics [26]. These cost-related barriers may be less pronounced in a system such as VHA, that utilizes the patient aligned care team (PACT) model where clinical pharmacists are systemically assigned to each PACT/PCP [51, 52]. It is notable that we found no significant differences in the reported utilization of clinical pharmacists among clinical sites (UH, AC, and VHA) and our findings suggest that the perceptions of the clinical pharmacist role have an influence on the limited utilization of pharmacist co-management.

This report is the first to broadly assess provider usage and preferences regarding clinical pharmacist support and other strategies in HTN management. However, the work has several limitations. First, the survey response rate of 37% introduces the possibility of response bias, as those who participated in the survey may not fully represent the attitudes and practices of all providers in this healthcare system. Second, self-reported survey responses can introduce recall bias or incorrect estimations in provider responses with regards to HTN management. While the healthcare systems from which the sample was drawn report blood pressure data, this is not available at the level of the clinic, which limits the ability of our study to directly show differences between percentages of controlled and uncontrolled hypertension in patient groups using CPPs compared to those who do not use CPP support. Finally, sample size can also be considered a limitation, particularly with respect to the smaller proportion of specialists participating in the survey compared to PCPs.

## Conclusion

Hypertension remains a significant public health challenge requiring innovative and collaborative approaches to improve patient outcomes. Our findings demonstrate underutilization of clinical pharmacists in hypertension management, despite strong evidence supporting their role in improving BP control, medication adherence, and satisfaction among both patients and providers. Addressing barriers to pharmacist integration and promoting their value in chronic disease medication management are essential to improve clinical outcomes in HTN patients. By leveraging the expertise of pharmacists, healthcare systems can optimize HTN management and meet the growing demands of an increasingly burdened healthcare environment.

## Data Availability

The datasets used and analyzed for the current study are available to researchers interested in further analyses and interpretation upon reasonable request. Please contact the corresponding author for inquiries regarding requests for data.

## Abbreviations

AC: Ambulatory Clinic
ACC: American College of Cardiology
BP: Blood Pressure
CAPTION: Collaboration Among Pharmacists and Physicians to Improve Outcome Now trial
CFR: Code of Federal Regulations
CKD: Chronic Kidney Disease
DM: Diabetes Mellitus
HTN: Hypertension
OHRP: Office for Human Research Protections
PACT: Patient Aligned Care Team
PCP: Primary Care Provider
TASMINH2: Telemonitoring and Self-Management in the Control of Hypertension trial
TASMIN-SR: Targets and Self-Management for the Control of Blood Pressure in Stroke and Other At-Risk Groups trial
UH: University Hospital
UIIRB: University of Iowa Institutional Review Board
VA: Veterans Affairs
